# DICER, DROSHA and DNA damage response RNAs are necessary for the secondary recruitment of DNA damage response factors

**DOI:** 10.1242/jcs.182188

**Published:** 2016-04-01

**Authors:** Sofia Francia, Matteo Cabrini, Valentina Matti, Amanda Oldani, Fabrizio d'Adda di Fagagna

**Affiliations:** 1 IFOM Foundation – The FIRC Institute of Molecular Oncology Foundation, Via Adamello 16, Milan 20139, Italy; 2 Istituto di Genetica Molecolare, Consiglio Nazionale delle Ricerche (IGM-CNR), Via Abbiategrasso 207, Pavia 27100, Italy

**Keywords:** DICER, DNA damage response, DROSHA, Non-coding RNA

## Abstract

The DNA damage response (DDR) plays a central role in preserving genome integrity. Recently, we reported that the endoribonucleases DICER and DROSHA contribute to DDR activation by generating small non-coding RNAs, termed DNA damage response RNA (DDRNA), carrying the sequence of the damaged locus. It is presently unclear whether DDRNAs act by promoting the primary recognition of DNA lesions or the secondary recruitment of DDR factors into cytologically detectable foci and consequent signal amplification. Here, we demonstrate that DICER and DROSHA are dispensable for primary recruitment of the DDR sensor NBS1 to DNA damage sites. Instead, the accumulation of the DDR mediators MDC1 and 53BP1 (also known as TP53BP1), markers of secondary recruitment, is reduced in DICER- or DROSHA-inactivated cells. In addition, NBS1 (also known as NBN) primary recruitment is resistant to RNA degradation, consistent with the notion that RNA is dispensable for primary recognition of DNA lesions. We propose that DICER, DROSHA and DDRNAs act in the response to DNA damage after primary recognition of DNA lesions and, together with γH2AX, are essential for enabling the secondary recruitment of DDR factors and fuel the amplification of DDR signaling.

## Abstract (precis)

**Summary:** We show that DICER, DROSHA and DNA damage response RNAs are necessary for the secondary recruitment of DNA damage response factors but not essential for primary recognition of DNA lesions.

## INTRODUCTION

The DNA damage response (DDR) is a multistep cellular signaling cascade responding to the generation of a variety of DNA lesions such as DNA double-strand breaks (DSB). DNA ends exposed upon DSB generation are thought to be promptly detected by the specialized DNA damage sensor complex MRE11–RAD50–NBS1 (MRN), which recruits the apical DDR activator ATM, a protein kinase that, upon recruitment to DNA lesions, becomes auto-phosphorylated and activated (Stracker and Petrini, 2011). Such direct recognition of DNA lesions is commonly referred to as primary recruitment (Celeste et al., 2003; Lukas et al., 2004). To amplify local DDR signaling at the site of DNA damage, activated ATM phosphorylates the histone variant H2AX *in cis* on Ser139 to form γH2AX. This is a key step in signal amplification because this histone mark leads to the recruitment of the DDR mediator proteins MDC1 and 53BP1 (also known as TP53BP1) (Bekker-Jensen et al., 2005; Melander et al., 2008; Chapman and Jackson, 2008), which in turn recruit more MRN–ATM complexes by protein–protein interactions (Ciccia and Elledge, 2010; Shiloh, 2006). This establishes an effective positive feedback loop that, by favoring the spreading of γH2AX from the DSB, leads to the so-called secondary recruitment of DDR factors to the damaged genomic locus, leading to the formation of cytologically detectable DDR foci (Lukas et al., 2011). Using H2AX knockout cells, it has been demonstrated that H2AX is not essential for the initial recognition of DNA breaks by DDR sensors such as NBS1 (also known as NBN) (primary recruitment), while γH2AX is essential for the secondary response to DNA damage that leads to the accumulation of DDR mediators such as MDC1 and 53BP1 in the form of DDR foci (secondary recruitment) (Celeste et al., 2002; Celeste et al., 2003; Fernandez-Capetillo et al., 2004). To date, no other signals independent of γH2AX have been reported to be necessary for DDR focus formation.

Factors of the RNA interference (RNAi) machinery have been recently shown to be present and active in the nucleus of mammalian cells (Gagnon et al., 2014) where they perform important functions in the regulation of chromatin, gene expression and splicing (Gonzalez et al., 2008; Ameyar-Zazoua et al., 2012; Gromak et al., 2013; White et al., 2014). We and others recently proposed that the RNAi-related endoribonucleases DICER and DROSHA promote DDR activation by generating small non-coding RNA (sncRNA) with the sequence of the DNA flanking the DSB (Francia et al., 2012; Wei et al., 2012; Gao et al., 2014; Michalik et al., 2012). In the context of DDR, we named them DNA damage response RNA (DDRNA) (Francia, 2015). We observed that DICER- or DROSHA-inactivated cells exposed to ionizing radiation show impaired DDR activation as demonstrated by reduced formation of DDR foci, impaired checkpoint and cellular senescence maintenance (Francia et al., 2012). Others have recently confirmed some of these observations (Kakarougkas et al., 2014; Wang and Goldstein, 2016). At present, at which stage of the DDR cascade DICER, DROSHA and their DDRNA products modulate DDR signaling is largely unknown. In this study, we aimed at distinguishing between their potential positive contribution to either the primary or the secondary recruitment of DDR factors to sites of DNA damage.

## RESULTS

### DICER or DROSHA knockdown does not affect the recruitment of NBS1–GFP to laser-induced DNA damage stripes while it reduces the recruitment of MDC1–GFP and 53BP1–GFP

To be able to identify a specific contribution to primary or secondary recruitment, we used two independent and complementary approaches: UVA laser micro-irradiation in BrdU pre-sensitized cells (Lukas et al., 2005) and a cell line carrying a cluster of inducible DSBs (Lan et al., 2010). By both approaches, differently from IR or radiomimetic drugs, the generation of numerous lesions densely packed in a defined nuclear space allows the detection and the study of the primary recruitment of DDR sensors to DNA lesions (Bekker-Jensen et al., 2005, 2006; Doil et al., 2009). With these two techniques, we tested if DICER or DROSHA inactivation impacts on the primary recognition of DNA lesions by NBS1, and/or on the secondary recruitment of MDC1 and 53BP1.

To study the recruitment of these DDR factors to DNA lesions in live cells, we used laser micro-irradiation in three separate U2OS cell lines expressing NBS1–GFP (Lukas et al., 2004), MDC1–GFP (Hable et al., 2012) or 53BP1–GFP (Bekker-Jensen et al., 2005) fusion proteins. The knockdown (KD) of DICER or DROSHA reduces the number of MDC1–GFP and 53BP1–GFP foci in these GFP reporter cell lines (Fig. S1A–C,F–H) as studied by treatment with neocarzinostatin (NCS), a radiomimetic drug that induces an acute dose of DSBs (Beerman and Goldberg, 1974). γH2AX foci are unaffected in the same cells (Fig. S1A,D,F,I) and the total levels of GFP fusion proteins are not diminished by DICER or DROSHA inactivation (Fig. S1E,J). These results confirm and extend our published observations (Francia et al., 2012) to DDR markers detectable as GFP fusion products and introduce an additional source of DNA damage (NCS), and they validate these cell lines for their use in identifying the involvement of DICER and DROSHA in modulating the primary or secondary response to DNA damage by laser micro-irradiation.

**Fig. 1.**
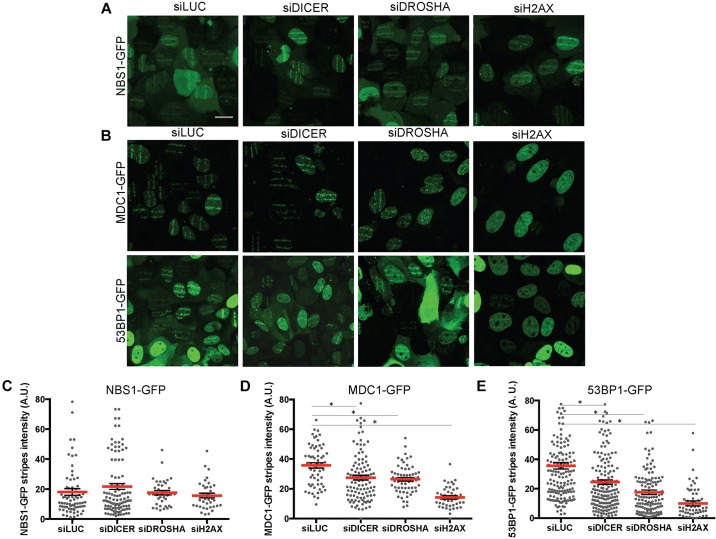
**DICER or DROSHA knockdown do not affect the recruitment of NBS1–GFP to laser stripes while reducing the recruitment of MDC1–GFP and 53BP1–GFP.** U2OS cells expressing NBS1–GFP, MDC1–GFP and 53BP1–GFP were transfected with siRNA against Luciferase (siLUC), DICER, DROSHA or H2AX. After 72 h, cells were laser-micro-irradiated and the recruitment of DDR–GFP fusion proteins was monitored at 20 min. (A,B) Representative images of NBS1–GFP (A), MDC1–GFP and 53BP1–GFP (B) cells exposed to micro-irradiation are shown. Scale bar: 20 μm. (C–E) Plots show the distribution of laser stripe fluorescence intensity of NBS1–GFP (C), MDC1–GFP (D) and 53BP1–GFP (E). Red bars indicate mean value, error bars indicate s.e.m. *P<0.01 by one-way ANOVA. For each condition shown 40–90 cells from three independent experiments were measured.

Since primary recruitment of NBS1 is H2AX independent (Celeste et al., 2003; Bekker-Jensen et al., 2006), NBS1–GFP detection on laser tracks is expected to be comparable in both control (siLuciferase-transfected) and siH2AX-transfected cells. Indeed, we observed that NBS1–GFP forms stripes at laser-induced DNA damage in both sets of cells, demonstrating that our experimental conditions show sufficient sensitivity in detecting the primary recruitment of NBS1 to DNA lesions in H2AX-KD cells (Fig. S2A). By contrast, MDC1–GFP and 53BP1–GFP secondary recruitment is expected to be impaired in the absence of H2AX (Bekker-Jensen et al., 2006) and indeed, whereas MDC1–GFP- and 53BP1–GFP-positive bright DNA damage-induced stripes were detectable in control cells, their formation was strongly reduced in H2AX-KD cells (Fig. S2B,C). Having established a clear readout of primary versus secondary recruitment, we studied the impact of DICER or DROSHA KD (Fig. S2D) on NBS1–GFP laser-induced DNA damage stripes formation. H2AX-KD cells were used as a control. We observed that NBS1–GFP recruitment to laser stripes is unaffected upon DICER, DROSHA or H2AX KD (Fig. 1A), indicating that they are not essential for NBS1 primary recruitment to DNA damage sites.

To provide quantitative analyses of these results, we analyzed images taken 20 min after laser micro-irradiation and measured the fluorescence intensity in the region exposed to laser-induced DNA damage in all conditions studied. These measurements (40–90 cells per point from three independent experiments) confirmed that NBS1–GFP recruitment is quantitatively unaltered in DICER- or DROSHA-KD cells (Fig. 1C).

Next, we analyzed MDC1–GFP and 53BP1–GFP recruitment to laser-induced DNA damage upon KD of DICER, DROSHA or H2AX (Fig. S2E). Excitingly, we observed that despite similar levels of BrdU incorporation to sensitize cells to laser treatment (Fig. S2F), MDC1–GFP and 53BP1–GFP stripes show a reduced intensity and a more discontinuous pattern in DICER- or DROSHA-KD cells (Fig. 1B). This visual evaluation was confirmed by the quantification of the fluorescence intensity of MDC1–GFP and 53BP1–GFP stripes of laser-induced DNA damage (Fig. 1D,E). Hence, similarly to γH2AX, DICER and DROSHA are required to establish a robust secondary recruitment of 53BP1 and MDC1 to sites of DNA damage.

### DICER or DROSHA inactivation does not affect the recruitment of endogenous NBS1 to laser-induced DNA damage stripes but it reduces the recruitment of endogenous MDC1 and 53BP1

**Fig. 2.**
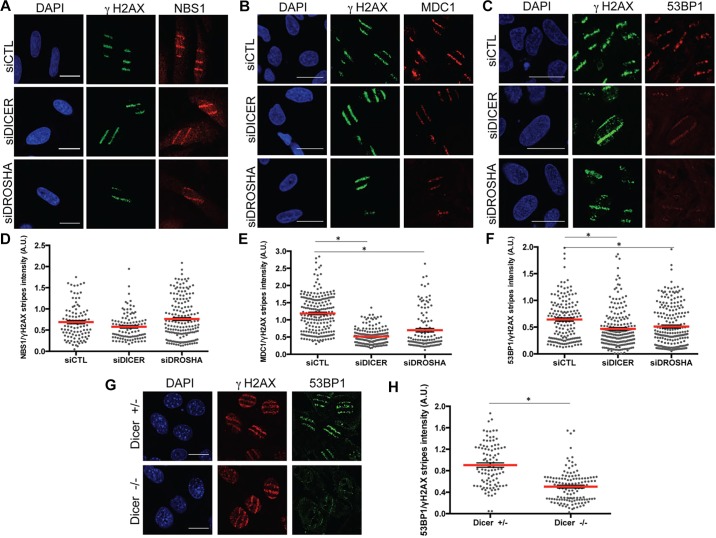
**DICER or DROSHA inactivations do not affect the recruitment of endogenous NBS1 to laser stripes but reduce the recruitment of endogenous MDC1 and 53BP1.** U2OS cells were knocked down for DICER, DROSHA or H2AX in the presence of 10 μM BrdU. After 72 h, cells were laser-micro-irradiated and then fixed 20 min later. (A–C) Laser stripe formation was monitored by indirect immunofluorescence and representative images are show for γH2AX and NBS1 (A), MDC1 (B) or 53BP1 (C). (D–F) Plots show the fluorescence intensity of NBS1 (D), MDC1 (E) or 53BP1 (F) laser stripes upon normalization of the fluorescence intensity to that of γH2AX in the same area. Red bars indicate mean value, error bars indicate s.e.m. *P<0.05 by one-way ANOVA. For each condition shown more than 20 cells from two independent experiments were analyzed. (G) Heterozygous *Dicer1*^+/−^ mouse sarcoma cell lines and knockout *Dicer1*^−/−^ cells were laser-micro-irradiated and fixed 20 min afterwards. Laser stripe formation was monitored by indirect immunofluorescence and representative images of γH2AX and 53BP1 laser-induced stripes are shown. Scale bars: 20 μm. (H) Plot showing the fluorescence intensity of 53BP1 laser stripes on normalization on the fluorescence intensity of γH2AX in the same area. Red bars indicate mean value, error bars indicate s.e.m. *P<0.001 by unpaired Student's *t*-test. For each condition shown more than 30 cells from two independent experiments were measured.

Next, we extended these analyses to endogenous DDR factors. So, DICER or DROSHA were knocked down by siRNA (Fig. S3A) in U2OS cells. Cell cycle progression was monitored by FACS analyses and excluded significant alterations upon DICER and DROSHA KD (Fig. S3B). Pre-sensitized cells were exposed to laser micro-irradiation, fixed 20 min later and stained with antibodies against endogenous γH2AX, MDC1 and 53BP1. In this setup, γH2AX staining was used to identify the nuclear region hit by the laser path and as a reference for the amount of DNA damage generated. Also in this setting, we observed that recruitment of endogenous NBS1 to sites of DNA damage was unaltered in DICER- and DROSHA-inactivated cells (Fig. 2A), while the accumulation of endogenous MDC1 (Fig. 2B) and 53BP1 (Fig. 2C) was reduced in DICER- or DROSHA-KD cells, compared with control siLuciferase-transfected cells. An accurate quantification confirmed these observations (Fig. 2D–F). Thus, DICER and DROSHA are not essential for the primary recruitment of endogenous NBS1 to sites of DNA damage, being required rather for the enforcement of the secondary response to DNA damage that leads to the accumulation of MDC1 and 53BP1.

The role played by DICER in DDR activation has not yet been studied in cells genetically null for this gene, as complete DICER loss was considered to be incompatible with somatic cell proliferation (Fukagawa et al., 2004; Kumar et al., 2009; Nittner et al., 2012; Arrate et al., 2010). However, the generation of a proliferating somatic mouse sarcoma cell line (*KrasG12D; Trp53^−/−^; Dicer1^f/−^*) in which *Dicer1* can be deleted by Cre–lox recombination has been recently reported (Ravi et al., 2012). Therefore, we decided to recapitulate our observations in this cell system (Fig. S3C–F). We observed that upon NCS treatment, formation of 53BP1 foci was reduced in *Dicer1*^−/−^ cells compared with *Dicer1*^+/−^ cells, whereas γH2AX accumulation was unaffected (Fig. S3C–E). Moreover, in pre-sensitized and laser-micro-irradiated cells, endogenous 53BP1 recruitment to γH2AX laser stripes was dramatically impaired in *Dicer1*^−/−^ cells (Fig. 2G,H), despite unaltered 53BP1 protein levels in *Dicer1*^−/−^ cells with respect to *Dicer1*^+/−^ cells (Fig. S3F).

Taken together, these results obtained by laser micro-irradiation in two independent cell lines indicate that primary recruitment of DDR sensors is independent of DICER and DROSHA, as well as H2AX, and they point to a role for DICER and DROSHA in fueling the secondary recruitment of DDR mediator proteins to sites of DNA damage, thus promoting signal amplification.

### DICER or DROSHA inactivation reduce the recruitment of endogenous MDC1 and 53BP1, but not of NBS1, to an I-SceI-induced cluster of DSBs

**Fig. 3.**
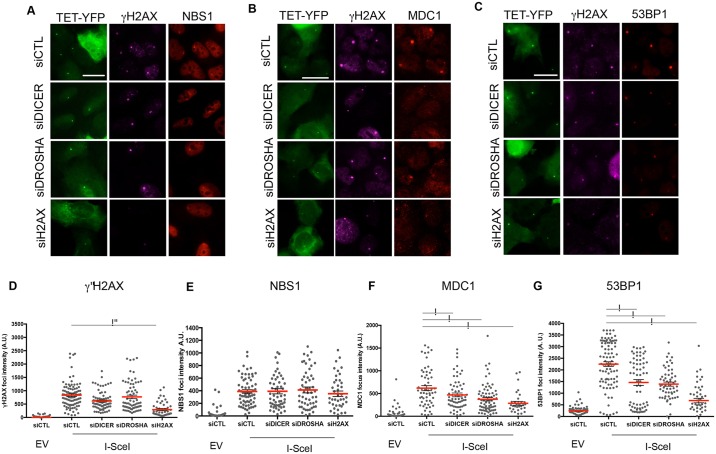
**DICER or DROSHA inactivation reduces the recruitment of endogenous MDC1 and 53BP1, but not NBS1, to I-SceI-induced DSB clusters.** DICER, DROSHA or H2AX were knocked down in U2OS/TRE/I-SceI-19 cells. After 48 h, cells were transfected with plasmids expressing TET–YFP and I-SceI. TET–YFP recruitment to the cut locus was induced the following day by doxycycline administration for 3 h. (A–C) DDR focus formation at clustered DSBs was monitored by indirect immunofluorescence for γH2AX and NBS1 (A), MDC1 (B) and 53BP1 (C). Representative images for I-SceI-induced γH2AX, NBS1, MDC1 or 53BP1 foci co-localizing with TET–YFP positive loci are shown. Scale bars: 20 μm. (D–G) Plots showing the quantification of the fluorescence intensity of γH2AX (D), NBS1 (E), MDC1 (F) or 53BP1 (G) foci at TET–YFP loci. Red bars indicate mean value, error bars indicate s.e.m. *P<0.05 by one-way ANOVA. For each condition shown more than 30 cells from two independent experiments were measured.

Laser micro-irradiation has been shown to generate a variety of different types of DNA lesions and, to an extent, also RNA and protein damage (Dinant et al., 2007). Therefore, to complement and strengthen our conclusions, we decided to expand our analyses with a different approach that allows the study of primary recruitment without employing laser light. We used the already established cell line U2OS/TRE/I-SceI-19 (Lan et al., 2010) that harbors the stable integration in a single locus of 200 copies of an array containing the cleavage site for the I-SceI endonuclease, flanked by tetracycline response elements (TREs) (TET repeats). The expression of TET–YFP and I-SceI allows the generation of up to 200 DSBs in close proximity and the formation of a large DDR focus co-localizing with the TET–YFP focal signal in the nucleus of each cell. This cellular system has been previously shown to allow the visualization of those DDR factors, such as Ku, that associate to DSBs in very few copies (Lan et al., 2010). These cells were therefore knocked down for DICER, DROSHA or H2AX (Fig. S4A), transfected with TET–YFP- and I-SceI-expressing plasmids, fixed 24 h later and stained with antibodies against γH2AX, NBS1, MDC1 and 53BP1. As expected, these DDR markers accumulated at the TET–YFP locus upon DSB induction by I-SceI (Fig. 3A–C) and, by quantifying the fluorescence intensities, we confirmed that γH2AX foci were significantly reduced in H2AX-KD cells but not in any other KD samples (Fig. 3D). Similarly, NBS1 foci were not reduced in DICER-, DROSHA- or H2AX-KD cells (Fig. 3A,E) indicating that the detection of NBS1 at the DSB cluster was the result of primary recruitment. By contrast, MDC1 and 53BP1 foci intensities showed a significant reduction in both DICER- and DROSHA-KD samples (Fig. 3B,C,F,G), as well as in H2AX-KD cells, as expected. Therefore, also in this system, DICER and DROSHA control the secondary recruitment of DDR factors while not affecting the recruitment of DNA damage sensors such as NBS1.

### NBS1 foci are resistant to RNaseA treatment whereas 53BP1 foci are sensitive to it and can be restored by incubation with sequence-specific synthetic DDRNAs

**Fig. 4.**
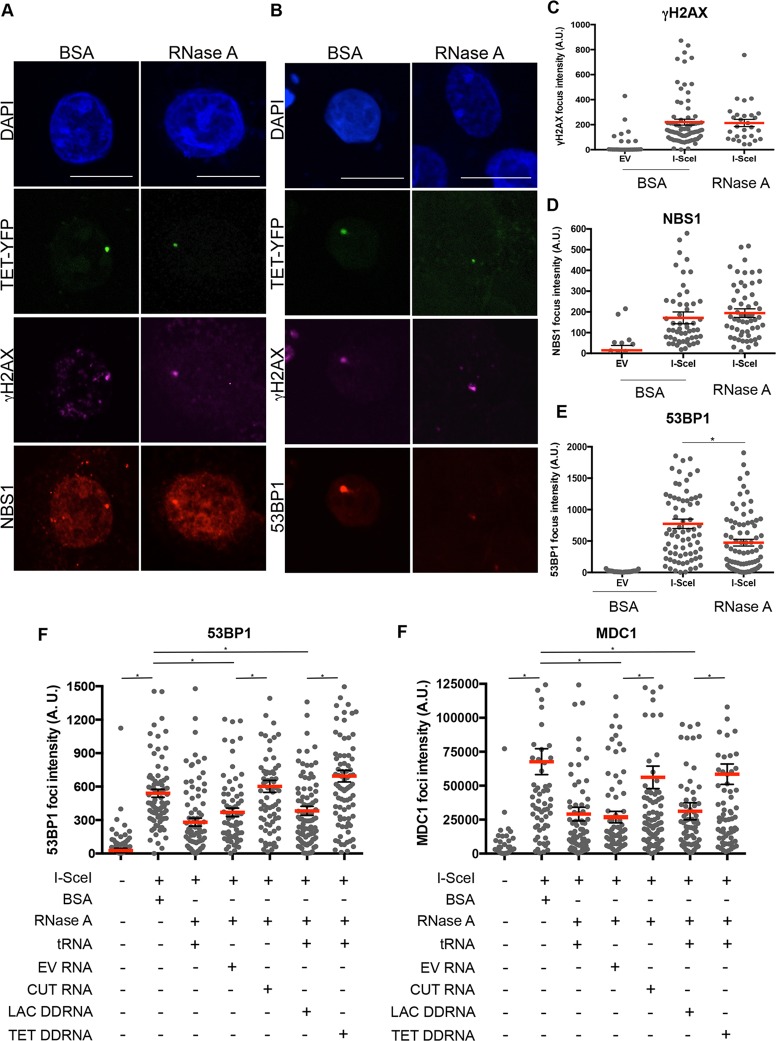
**NBS1 foci are resistant to RNaseA treatment, whereas 53BP1 and MDC1 foci are sensitive to it and can be restored by incubation with RNAs from cells with cut DNA or sequence-specific synthetic DDRNAs.** U2OS/TRE/I-SceI-19 cells were transfected with plasmids expressing TET–YFP and I-SceI, and TET–YFP recruitment to the cut locus was induced the following day by doxycycline administration for 3 h. Cells were mildly permeabilized and treated with RNaseA, or BSA as control, and focus formation at clustered DSBs was probed with antibodies against γH2AX and NBS1 or 53BP1. (A,B) Representative images for I-SceI-induced γH2AX and NBS1 (A) or 53BP1 (B) foci co-localizing with TET–YFP-positive loci after treatment with BSA or RNaseA are shown. Scale bars: 20 μm. (C–E) Plots showing the quantification of the fluorescence intensity of γH2AX (C), NBS1 (D), 53BP1 (E) foci at TET–YFP loci. Red bars indicate mean value, error bars indicate s.e.m. *P<0.05 by one-way ANOVA. For each condition shown more than 40 cells from two independent experiments were measured. (F,G) Plots showing the quantification of the fluorescence intensity of 53BP1 (F) or MDC1 foci (G) at TET–YFP loci after treatment with BSA or RNaseA and complementation with different RNAs. 53BP1 and MDC1 foci are restored post-RNaseA treatment only when cells are incubated with RNA from cells with cut DNA (CUT RNA) or specific synthetic RNA (TET), and not if cells are incubated with yeast tRNA, RNA from cells with uncut DNA transfected with an empty expression vector (UNCUT RNA) or synthetic RNA with an unrelated sequence (LAC). *P<0.05 by one-way ANOVA. For each condition shown more than 40 cells from two independent experiments were measured.

We previously demonstrated that the DDR-related function of DICER is dependent on its endoribonuclease activity and on its role in DDRNA biogenesis (Francia et al., 2012). Moreover, in agreement with Pryde et al., (2005), we observed that 53BP1 foci forming at individual DSBs disassemble upon RNaseA treatment (Francia et al., 2012). We further showed that following RNaseA removal, 53BP1 foci can reform if cells are incubated with RNA purified from cells in which DNA was cut by the activity of I-SceI, or with chemically synthesized locus-specific DDRNAs (Francia et al., 2012). This observation demonstrates a direct contribution of DDRNA to DDR focus formation, independent of canonical RNA interference mechanisms; nevertheless, it cannot distinguish between their specific contribution to primary or secondary recruitment. This is because in the cellular systems used, DDR is activated at individual DNA lesions and ensuing DDR foci are detectable only if both primary and secondary recruitment take place. Therefore, we decided to test the impact of RNaseA treatment on detectable NBS1 and 53BP1 foci (markers of primary and secondary recruitment, respectively) forming at the DSBs cluster in U2OS/TRE/I-SceI-19 cells. With this aim, TET–YFP-expressing cells containing DNA cut with I-SceI were permeabilized and treated with RNaseA, or BSA as a control. After fixation and staining for NBS1 or 53BP1, we monitored sensitivity of DDR foci to RNaseA (Fig. 4B,C). γH2AX staining in the same cells was used as a reference for DNA damage induction and chromatin integrity (Fig. 4A–C). We observed that NBS1 accumulation to the I-SceI-induced DDR focus was comparable in BSA- or RNaseA-treated cells (Fig. 4A,D), indicating that NBS1 recruitment is not dependent on RNA. By contrast, 53BP1 accumulation was eminently sensitive to RNaseA treatment (Fig. 4B,E). This result suggests that 53BP1 secondary recruitment to the DSB cluster requires RNAs.

Since primary recruitment of NBS1 is not dependent on RNA, we reasoned that this system is the best experimental setup to clearly demonstrate that DDRNA act by promoting secondary recruitment. We thus isolated total RNA from empty-vector-transfected cells with uncut DNA (UNCUT RNA) or I-SceI-expressing cells with cut DNA (CUT RNA) and we tested the activity of these two RNA preparations in DDR focus reformation post-RNaseA treatment. Quantification of the fluorescence intensity of 53BP1 or MDC1 foci at the TET–YFP-labeled genomic locus shows that 53BP1 and MDC1 secondary recruitment was reduced in cells treated with RNaseA and complemented with inert tRNA (Fig. 4F,G). Similarly, incubation of RNaseA-treated cells with total RNA from cells with uncut DNA (UNCUT RNA) did not restore focus formation, confirming that DDRNAs accumulate upon damage. By contrast, 53BP1 and MDC1 secondary recruitment was completely restored in RNaseA-treated cells by incubation with equal amount of RNA coming from cells with cut DNA (CUT RNA) (Fig. 4F,G). Finally, to confirm that the RNA required for focus restoration is sequence specific, we took advantage of the fact that in the engineered locus of U2OS/TRE/I-SceI-19 cells DSBs are flanked by an array of TET repeats, and we used the already validated synthetic small RNAs with TET-repeat sequences (Francia et al., 2012). We compared their ability to allow 53BP1 and MDC1 focus re-formation at the DSB cluster with that of control DDRNAs with a LAC operon sequence, absent in this cellular system. Fluorescence intensity of 53BP1 and MDC1 foci could be restored only by incubation with sequence-specific synthetic TET DDRNAs but not with control synthetic LAC DDRNA (Fig. 4F,G). As previously observed, γH2AX focus intensity also remained unaltered in this system (Fig. S4B). By this *in situ* approach we could therefore demonstrate that the secondary recruitment of 53BP1 to a cluster of DNA damage sites is directly controlled by sequence-specific DDRNAs.

## DISCUSSION

In summary, here we investigate for the first time the potential contribution of DICER, DROSHA and DDRNAs to the direct recognition of DNA lesions by DNA damage sensors such as the MRN complex and to the so-called secondary recruitment of DDR factors. By the use of two independent experimental setups, we demonstrate that DICER, DROSHA and DDRNAs do not impact on direct sensing of DNA lesions. This is a feature reminiscent of the role of H2AX (Celeste et al., 2003) and suggests that DDRNA are unlikely to directly interact with DSB DNA ends, despite their sequence homology. Instead, we observed that DICER and DROSHA are required for the secondary recruitment of DDR factors in two experimental settings where a high number of DNA lesions are densely formed in the same locus. We therefore propose that for an efficient DDR focus formation and thus an effective DDR signal amplification, at least two events must occur; γH2AX formation and generation of DDRNA by DICER and DROSHA. These are likely parallel pathways as DICER and DROSHA are dispensable for H2AX phosphorylation (Francia et al., 2012; Wei et al., 2012; Gao et al., 2014).

MDC1 binds directly to γH2AX and it is thought to nucleate focus formation (Stucki et al., 2005). The observation that MDC1 recruitment can be uncoupled from the accumulation of γH2AX upon DICER or DROSHA inactivation or RNaseA treatment indicates that MDC1 binding to γH2AX, although necessary (Lukas et al., 2004; Stucki et al., 2005; Melander et al., 2008; Jungmichel et al., 2012), might not be sufficient in an *in vivo* setting to guarantee stable MDC1 association to γH2AX foci.

The observation that DDRNAs act in parallel with γH2AX in focus nucleation suggests that DDRNA recruitment to the damaged genomic locus must occur. It is tempting to speculate that DDRNA might act together with γH2AX in nucleating the large structures known as DDR foci by providing a scaffold of RNA–protein interactions (Jankowsky and Harris, 2015) that retain DDR factors in proximity to DNA lesions. The functions described here of DICER, DROSHA and DDRNAs in enabling the secondary recruitment of DDR factors point to a role of DDRNAs in coordinating focus formation and signal amplification following the primary recognition of the lesion.

## MATERIALS AND METHODS

### Cultured cells and treatments

U2OS 53BP1–GFP (Bekker-Jensen et al., 2005), U2OS NBS1–GFP (Lukas et al., 2004; both a kind gift from Jiri Bartek), U2OS/TRE/I-SceI-19 (Lan et al., 2010; a kind gift from Prof. A. Yasui) were grown in DMEM supplemented with 10% fetal bovine serum, 1% L-glutamine, plus 1% penicillin/streptomycin and G418 (250 µg/ml), Puromycine (1 µg/ml) or Zeocin (250 µg/ml), respectively. U2OS MDC1–GFP cells (Hable et al., 2012, a kind gift from Guido Drexler) were grown in RPMI 1640, supplemented with 10% fetal bovine serum, 1% L-glutamine, 1% penicillin/streptomycin and puromycin (0.8 µg/ml). *KrasG12D; Trp53^−/−^; Dicer1^f/−^* and *KrasG12D; Trp53^−/−^; Dicer1^−/−^* cells (Ravi et al., 2012) were grown in DMEM, 10% fetal bovine serum, 1% L-glutamine and 1% penicillin/streptomycin. DNA damage was generated by treatment with 50 ng/ml Neocarzinostatin (NCS; Sigma, N9162) for 20 min at 37°C. All cell lines are routinely screened against mycoplasma contamination.

### Antibodies

Mouse monoclonal anti-γH2AX (Ser139) (clone JBW301; 1:500; Millipore, 05-636); rabbit polyclonal anti-γH2AX (Ser139) (1:300; Novus Biological, NB100-384); rabbit polyclonal anti-γH2AX (Ser139) (1:500; Abcam, ab11174); rabbit polyclonal anti-NBS1 (1:300; Novus Biological, NB100-143); rabbit polyclonal anti-53BP1 (1:1000; Novus Biological, NB100-304); rabbit polyclonal anti-53BP1 (1:1000; Bethyl A300-272A); mouse monoclonal anti-MDC1 (clone mdc1-50; 1:400; Sigma, M2444); sheep polyclonal anti-MDC1 (#3035; 1:400; kind gift from Raimundo Freire; Hospital Universitario de Canarias, Instituto de Tecnologías Biomédicas, La Laguna, Tenerife, Spain); mouse monoclonal anti-BrdU (clone B44; 1:100; Beckton Dikinson, 347580); rabbit polyclonal anti-GFP (1:1000; Life Technologies, A111-22); mouse monoclonal anti-Vinculin (clone 7F9; 1:5000; Millipore, MAB3574); rabbit polyclonal anti-Dicer1 (1:500; Sigma, HPA00694); rabbit monoclonal anti-Drosha (clone D28B1; 1:1000; Cell Signaling, 3364).

### Indirect immunofluorescence

Cells were plated on coverslips or in glass-bottom dishes Mattek (P35G-1.5-14-C). Staining for NBS1, MDC1, 53BP1, γH2AX and BrdU were performed as described previously (Di Micco et al., 2006). Cells were fixed in 4% paraformaldehyde followed by a 0.2% Triton X-100 permeabilization step if expressing a GFP or a YFP reporter gene or methanol:acetone 1:1 in all other cases. Coverslips and glass-bottom dishes were incubated for 1 h in 1× PBG blocking buffer [0.5% BSA, 0.2% gelatin from cold-water fish skin (Sigma-Aldrich, G7765) in 1× PBS]. Incubation with primary antibody, diluted in 1× PBG blocking buffer, was for 1 h at room temperature. Coverslips were washed three times with 1× PBG buffer and incubated with secondary antibody diluted in 1× PBG blocking buffer for 40 min at room temperature. Coverslips and glass-bottom dishes were extensively washed two times in 1× PBG blocking buffer and two times in 1× PBS. Nuclei were stained with 0.2 µg/ml DAPI (4′,6-diamidino-2-phenylindole; Sigma-Aldrich, D9564) in 1× PBS for 1 min at room temperature and coverslips were mounted in 1,4-Diazabicyclo[2.2.2]octane and incubated at room temperature overnight in the dark. After DAPI staining, glass-bottom dishes were incubated for 5 min with 4% PFA, washed and kept in PBS at 4°C. Images were acquired using a Leica TCS SP2 AOBS confocal system equipped with a Leica HCX PL APO 63×/1.4NA oil immersion objective and the Leica Confocal Software (LCS), keeping the Lookup Table (LUT) in a linear range using an oil immersion objective or with a widefield epifluorescence microscope (Olympus IX71) equipped with PlanApo 60×/1.40NA oil immersion objective, a Cool SNAP ES camera (Photometrics) and driven by MetaMorph software (Universal Imaging Corporation). Comparative immunofluorescence analyses were performed in parallel with identical acquisition parameters and imaging analyses were performed using ImageJ (NIH) and CellProfiler (Carpenter et al., 2006) software.

### Immunoblotting

Cells were lysed in Laemmli sample buffer [2% sodium dodecyl sulphate (SDS), 5% glycerol, 1.5% DTT, 0.01% bromophenol blue, 60 mM Tris HCl pH 6.8]. Collected cells were sonicated (Braun) with three bursts of 15 s and heated for 10 min at 95°C. A 6% SDS-polyacrylamide gel with a width of 1 mm was loaded with 15 μl of lysate, along with 6 µl of molecular weight markers (BioRad). Gels were run in Tris-glycine electrophoresis buffer (25 mM Tris, 250 mM glycine, 0.1% SDS) until the dye reached the bottom of the gel. For western blotting analysis proteins were transferred to a 0.2 µm nitrocellulose membrane (BioRad Trans-Blot Turbo transfer pack) using the Trans-Blot Turbo Transfer System apparatus (BioRad). The transfer was performed at 25 V for 10 min. Membranes were incubated with 5% skim milk in TBS-Tween buffer for 1 h, followed by overnight incubation at 4°C with primary antibody and three washes with TBS-Tween before 1 h incubation at room temperature with the specific HRP-conjugated secondary antibody. Chemiluminescence detection was done by incubation with Luminata Classico or Crescendo (Millipore). Proteins were visualized by autoradiography on ECL films (Amersham), using various exposure times and manually developed.

### Plasmids

TET–YFP (pEYFP-rtTA-N1), generated in the laboratory of D.L. Spector, -SceI plasmid was a kind gift from Dr Yasui (Lan et al., 2010); the empty vector used was pCMV5 (ATCC-87781).

### siRNA

The DHARMACON siRNA were transfected by Lipofectamine RNAi Max (Invitrogen) at a final concentration of 20 nM. Sequences for human DICER, DROSHA and H2AX, siLuciferase and non-targeting sequences are given in Table 1.

**Table 1.**
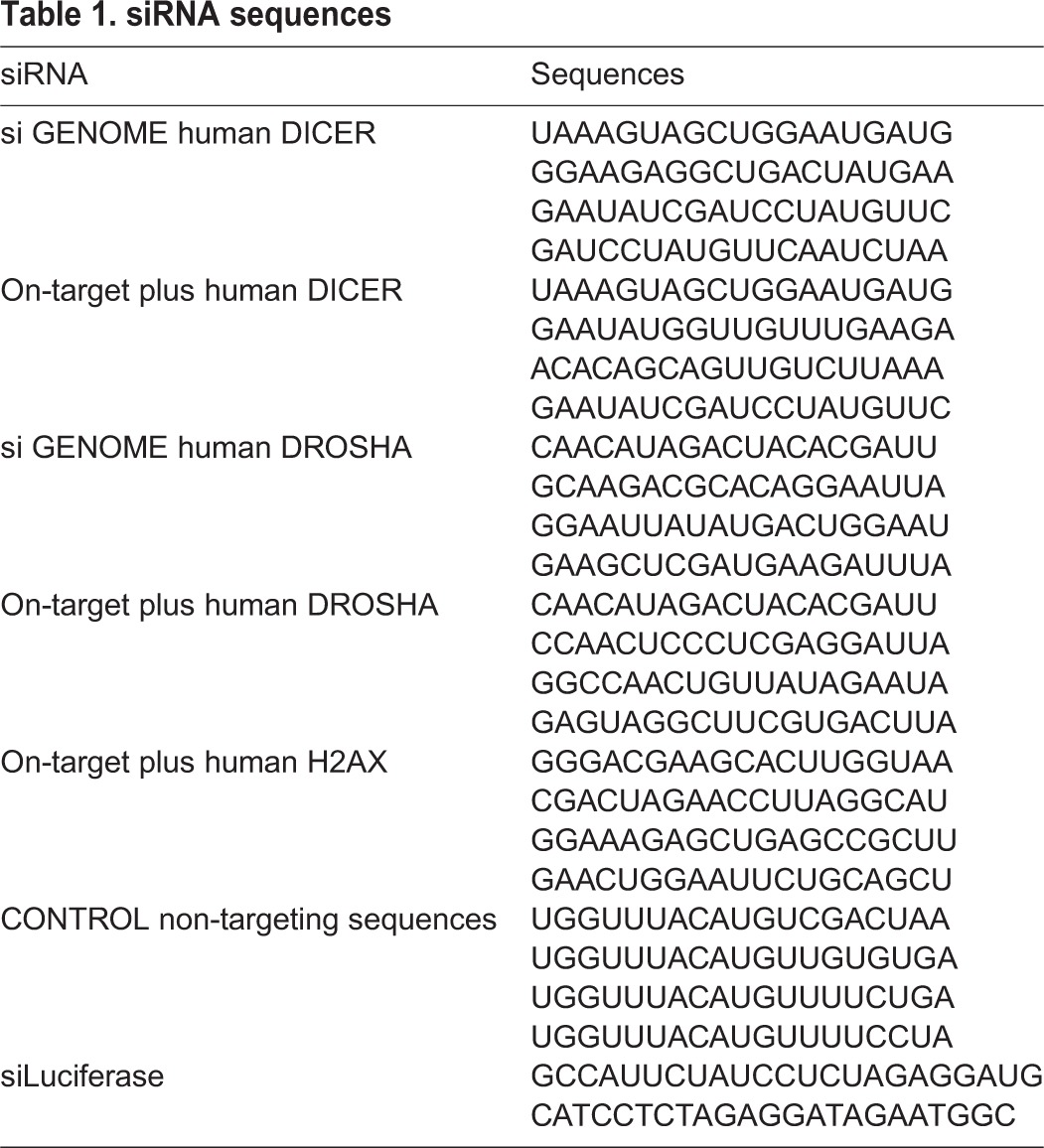
**siRNA sequences**

### Real-time quantitative PCR

Total RNA was isolated from cells using an RNeasy kit (Qiagen) according to the manufacturer's instructions and treated with DNase (Life Technologies) before reverse transcription. cDNA was generated using Superscript III Reverse Transcriptase or SuperScriptVILO (Invitrogen) and used as a template in real-time quantitative PCR (RT-qPCR) analysis. RT-qPCR reactions were performed on a Roche LightCycler 480 Sequence Detection System. The reactions were prepared using SYBR Green reaction mix (Qiagen, Roche). Beta-2-microglobulin (B2M) or ribosomal protein P0 (RPLP0) were used as human housekeeper genes, and mouse ribosomal protein P0 (RPLP0) was used as a mouse housekeeper gene.

#### Primer sequences for real-time quantitative PCR

Human and mouse RPLP0, F: 5′-TTCATTGTGGGAGCAGAC, R: 5′-CAGCAGTTTCTCCAGAGC; Human B2M, F: 5′-TTCTGGCCTGGAGGCTATC, R: 5′-TCAGGAAATTTGACTTTCCATTC; Human DICER, F: 5′-TGTTCCAGGAAGACCAGGTT, R: 5′-ACTATCCCTCAAACACTCTGGAA; Human DROSHA, F: 5′-GGCCCGAGAGCCTTTTATAG, R: 5′-TGCACACGTCTAACTCTTCCAC; Mouse DICER, F: 5′-GCAAGGAATGGACTCTGAGC, R: 5′-GGGGACTTCGATATCCTCTTC.

### Laser-induced DNA damage

Live-cell imaging and laser-induced DNA damage were performed on a Leica TCS SP5 point scanning confocal microscope equipped with a Leica HCX PL APO 63×/1.4NA oil immersion objective and an environmental microscope incubator (OKOLab) set to 37°C and 5% CO_2_ perfusion. The Leica TCS SP5 confocal microscope was driven by Leica LAS AF software. Cells were cultured in glass-bottom dishes (Mattek P35G-1.5-14-C) and pre-sensitized for 72 h in 10 µm BrdU. Laser micro-irradiation was carried out using a 50 mW 405 nm diode laser with a 100% power output. At 2× digital magnification, multiple regions of interest (ROI) of the same size were selected in each nucleus and the 405 nm laser was used to scan the ROIs for 50 iterations (total dwell time per pixel 490 μs).

### Cell-cycle analyses by FACS

U2OS cells were collected 72 h post transfection with siRNA, washed in PBS and fixed in 75% ethanol overnight at 4°C. 10^6^ fixed cells for each condition were washed once in PBS 1% BSA and re-suspended in PBS containing propidium iodide (50 μg/ml) and RNaseA (250 μg/ml), and incubated overnight in the dark. FACS analysis was performed on single cell suspensions. For each measurement, at least 20,000 cells were acquired. Samples were acquired on a FACSCanto II (Becton Dickinson). Propidium iodide was excited with a 488 nm laser and emission was detected with a 670LPnm filter. Data were acquired and analyzed with FACSDiva 6.1.1 (Becton Dickinson) and ModFit LT 3.0 (Verity Software House) software.

### RNaseA treatment and complementation with purified cellular RNA or DDRNA

U2OS/TRE/I-SceI-19 cells were seeded on 6-well plates, transfected by Lipofectamine 2000 transfection reagent (Invitrogen, 11668-027) with TET–YFP (0.7 µg) and I-SceI or empty (both 2 µg) plasmids, and 24 h later were incubated with doxycyclin (1 µg/ml) for 3 h. Cells were then permeabilized in 0.3% Tween20 (Euroclone) in PBS for 10 min at room temperature. After washing in PBS, cells were incubated for 20 min at room temperature with 0.2 µg/ml Ribonuclease A from bovine pancreas (Sigma-Aldrich) or acetylated BSA (0.2 µg/ml) in PBS+0.02 mM sodium acetate and 0.2 mM Tris-HCl pH 7.4. Cells were then washed extensively in sterile cold PBS and fixed in PFA 4%. For complementation with purified cellular RNA or DDRNAs U2OS/TRE/I-SceI-19 cells were seeded on coverslips and transfected with vectors expressing TET–YFP and I-SceI. After 24 h cells were permeabilized in 0.3% Tween20 (Sigma) in sterile PBS for 10 min at room temperature. After washing in sterile PBS, cells were pre-incubated in 70 μl of a solution containing PBS, 80 units of RNase inhibitor (RNaseOUT, Life Technologies, 40 units/μl) and alpha-amanitin (20 µg/ml) for 10 min. The coverslips were then transferred to 70 μl of the same solution of RNaseOUT and alpha-amanitin in PBS, complemented with 50 µg total RNA from cells with cut or uncut DNA, or same amount of yeast tRNA either alone or with annealed TET1 and TET2 DDRNAs (1 nM final concentration), or LAC1 and LAC2 as controls, for an additional 20 min at room temperature. Cells were then fixed in PFA 4% and stained for DDR markers as described. Synthetic DDRNA sequences: LAC1, P-AGC GGA UAA CAA UUU GUG GCC ACA UGU GGA; LAC2, P-UGU GGC CAC AAA UUG UU; TET1, P-ACU CCC UAU CAG UGA UAG AGA AAA GUG AAA GU; TET2, P-CUU UCA CUU UUC UCU AUC ACU GAU AGG GAG UG.

### Imaging analysis

In the case of micro-irradiation in NBS1–GFP, MDC1–GFP and 53BP1–GFP reporter cell lines, images were acquired with a Leica TCS SP5 confocal microscope equipped with a Leica HCX PL APO 63×/1.4NA oil immersion objective and driven by Leica LAS AF software. GFP signal in laser-damage-induced stripes were quantified by ImageJ by drawing the ROI of laser damage. The mean fluorescence intensity in each damaged area was measured and the mean intensity of an identical area in an undamaged region of the same nucleus was subtracted as background. For staining of endogenous proteins, images of stripes of laser-induced DNA damage were acquired with a Leica SP2 AOBS, equipped with Leica HCX PL APO 63×/1.4NA oil immersion objective and analyzed with the imaging analyses software CellProfiler (Carpenter et al., 2006), by using an ad-hoc-designed pipeline that creates a mask around the nuclear γH2AX-positive damaged area and identifies the same area in the corresponding pictures from other channels. The software measures the fluorescence intensity of γH2AX in that area and the fluorescence intensity of NBS1 or MDC1 or 53BP1 channels in the same nuclear region. Mean fluorescence intensity for each stripe of NBS1 or MDC1 or 53BP1 was expressed as a ratio with γH2AX fluorescence intensity. In the U2OS/TRE/I-SceI-19 system, images were acquired with a widefield epifluorescence microscope (Olympus IX71) equipped with PlanApo 60×/1.40NA oil immersion objective. Photomicrographs were taken with digital camera Cool SNAP ES (Photometrics) and data acquisition was done using MetaMorph software (Universal Imaging Corporation). DDR focus intensity was analyzed by ImageJ software by defining the ROI around the focus of TET–YFP signal. All the ROI defined were then localized in the MDC1 and 53BP1 channel and fluorescence intensity was measured in each ROI for each marker. From the integrated fluorescence density of each ROI we subtracted the integrated fluorescence density of an identical area in an undamaged region of the same nucleus, for all markers analyzed. Numbers of DDR foci per nucleus were quantified by the automated software CellProfiler, applying an ad-hoc-designed pipeline which, based on size and fluorescence intensity of DDR foci relative to the background signal, recognizes and counts their number in each DAPI-positive cell nucleus. Identical parameters were applied in the analyses of all conditions compared in each experiment. All data for imaging analyses were plotted with the use of GraphPad Prism software.

### Statistical analyses

Fluorescence intensity results are shown as means±standard error (s.e.m.). RT-qPCR results in the Supplementary information are shown as means of a technical triplicate±standard deviation (s.d.). *P*-values were calculated by non-parametric one-way ANOVA (because data distribution was negative using Shapiro–Wilk normality test) with multiple comparisons or unpaired Student's *t*-test using to GraphPad Prism statistics tools.

## References

[JCS182188C1] Ameyar-Zazoua M., Rachez C., Souidi M., Robin P., Fritsch L., Young R., Morozova N., Fenouil R., Descostes N., Andrau J. C. et al. (2012). Argonaute proteins couple chromatin silencing to alternative splicing. *Nat. Struct. Mol. Biol.* 19, 998-1004. 10.1038/nsmb.2373 22961379

[JCS182188C2] Arrate M. P., Vincent T., Odvody J., Kar R., Jones S. N. and Eischen C. M. (2010). MicroRNA biogenesis is required for Myc-induced B-cell lymphoma development and survival. *Cancer Res.* 70, 6083-6092. 10.1158/0008-5472.CAN-09-4736 20587524 PMC2906119

[JCS182188C3] Beerman T. A. and Goldberg I. H. (1974). DNA strand scission by the antitumor protein neocarzinostatin. *Biochem. Biophys. Res. Commun.* 59, 1254-1261. 10.1016/0006-291X(74)90449-5 4369938

[JCS182188C4] Bekker-Jensen S., Lukas C., Melander F., Bartek J. and Lukas J. (2005). Dynamic assembly and sustained retention of 53BP1 at the sites of DNA damage are controlled by Mdc1/NFBD1. *J. Cell Biol.* 170, 201-211. 10.1083/jcb.200503043 16009723 PMC2171401

[JCS182188C5] Bekker-Jensen S., Lukas C., Kitagawa R., Melander F., Kastan M. B., Bartek J. and Lukas J. (2006). Spatial organization of the mammalian genome surveillance machinery in response to DNA strand breaks. *J. Cell Biol.* 173, 195-206. 10.1083/jcb.200510130 16618811 PMC2063811

[JCS182188C6] Carpenter A. E., Jones T. R., Lamprecht M. R., Clarke C., Kang I. H., Friman O., Guertin D. A., Chang J. H., Lindquist R. A., Moffat J. et al. (2006). CellProfiler: image analysis software for identifying and quantifying cell phenotypes. *Genome Biol.* 7, R100 10.1186/gb-2006-7-10-r100 17076895 PMC1794559

[JCS182188C7] Celeste A., Petersen S., Romanienko P. J., Fernandez-Capetillo O., Chen H. T., Sedelnikova O. A., Reina-San-Martin B., Coppola V., Meffre E., Difilippantonio M. J. et al. (2002). Genomic instability in mice lacking histone H2AX. *Science* 296, 922-927. 10.1126/science.1069398 11934988 PMC4721576

[JCS182188C8] Celeste A., Fernandez-Capetillo O., Kruhlak M. J., Pilch D. R., Staudt D. W., Lee A., Bonner R. F., Bonner W. M. and Nussenzweig A. (2003). Histone H2AX phosphorylation is dispensable for the initial recognition of DNA breaks. *Nat. Cell Biol.* 5, 675-679. 10.1038/ncb1004 12792649

[JCS182188C9] Chapman J. R. and Jackson S. P. (2008). Phospho-dependent interactions between NBS1 and MDC1 mediate chromatin retention of the MRN complex at sites of DNA damage. *EMBO Rep.* 9, 795-801. 10.1038/embor.2008.103 18583988 PMC2442910

[JCS182188C10] Ciccia A. and Elledge S. J. (2010). The DNA damage response: making it safe to play with knives. *Mol. Cell* 40, 179-204. 10.1016/j.molcel.2010.09.019 20965415 PMC2988877

[JCS182188C11] Di Micco R., Fumagalli M., Cicalese A., Piccinin S., Gasparini P., Luise C., Schurra C., Garre M., Nuciforo P. G., Bensimon A. et al. (2006). Oncogene-induced senescence is a DNA damage response triggered by DNA hyper-replication. *Nature* 444, 638-642. 10.1038/nature05327 17136094

[JCS182188C12] Dinant C., de Jager M., Essers J., van Cappellen W. A., Kanaar R., Houtsmuller A. B. and Vermeulen W. (2007). Activation of multiple DNA repair pathways by sub-nuclear damage induction methods. *J. Cell Sci.* 120, 2731-2740. 10.1242/jcs.004523 17646676

[JCS182188C13] Doil C., Mailand N., Bekker-Jensen S., Menard P., Larsen D. H., Pepperkok R., Ellenberg J., Panier S., Durocher D., Bartek J. et al. (2009). RNF168 binds and amplifies ubiquitin conjugates on damaged chromosomes to allow accumulation of repair proteins. *Cell* 136, 435-446. 10.1016/j.cell.2008.12.041 19203579

[JCS182188C14] Fernandez-Capetillo O., Lee A., Nussenzweig M. and Nussenzweig A. (2004). H2AX: the histone guardian of the genome. *DNA Rep.* 3, 959-967. 10.1016/j.dnarep.2004.03.024 15279782

[JCS182188C15] Francia S. (2015). Non-coding RNA: sequence-specific guide for chromatin modification and DNA damage signaling. *Front. Genet.* 6, 807 10.3389/fgene.2015.00320 PMC4643122 26617633

[JCS182188C16] Francia S., Michelini F., Saxena A., Tang D., de Hoon M., Anelli V., Mione M., Carninci P. and d'Adda di Fagagna F. (2012). Site-specific DICER and DROSHA RNA products control the DNA-damage response. *Nature* 488, 231-235. 10.1038/nature11179 22722852 PMC3442236

[JCS182188C17] Fukagawa T., Nogami M., Yoshikawa M., Ikeno M., Okazaki T., Takami Y., Nakayama T. and Oshimura M. (2004). Dicer is essential for formation of the heterochromatin structure in vertebrate cells. *Nat. Cell Biol.* 6, 784-791. 10.1038/ncb1155 15247924

[JCS182188C18] Gagnon K. T., Li L., Chu Y., Janowski B. A. and Corey D. R. (2014). RNAi factors are present and active in human cell nuclei. *Cell Rep.* 6, 211-221. 10.1016/j.celrep.2013.12.013 24388755 PMC3916906

[JCS182188C19] Gao M., Wei W., Li M.-M., Wu Y.-S., Ba Z., Jin K.-X., Li M.-M., Liao Y.-Q., Adhikari S., Chong Z. et al. (2014). Ago2 facilitates Rad51 recruitment and DNA double-strand break repair by homologous recombination. *Cell Res.* 24, 532-541. 10.1038/cr.2014.36 24662483 PMC4011338

[JCS182188C20] Gonzalez S., Pisano D. G. and Serrano M. (2008). Mechanistic principles of chromatin remodeling guided by siRNAs and miRNAs. *Cell Cycle* 7, 2601-2608. 10.4161/cc.7.16.6541 18719372

[JCS182188C21] Gromak N., Dienstbier M., Macias S., Plass M., Eyras E., Cáceres J. F. and Proudfoot N. J. (2013). Drosha regulates gene expression independently of RNA cleavage function. *Cell Rep.* 5, 1499-1510. 10.1016/j.celrep.2013.11.032 24360955 PMC3898267

[JCS182188C22] Hable V., Drexler G. A., Brüning T., Burgdorf C., Greubel C., Derer A., Seel J., Strickfaden H., Cremer T., Friedl A. A. et al. (2012). Recruitment kinetics of DNA repair proteins Mdc1 and Rad52 but not 53BP1 depend on damage complexity. *PLoS ONE* 7, e41943 10.1371/journal.pone.0041943 22860035 PMC3408406

[JCS182188C23] Janicki S. M., Tsukamoto T., Salghetti S. E., Tansey W. P., Sachidanandam R., Prasanth K. V., Ried T., Shav-Tal Y., Bertrand E., Singer R. H. et al. (2004). From silencing to gene expression: real-time analysis in single cells. *Cell* 116, 683-698. 10.1016/S0092-8674(04)00171-0 15006351 PMC4942132

[JCS182188C24] Jankowsky E. and Harris M. E. (2015). Specificity and nonspecificity in RNA–protein interactions. *Nat. Rev. Mol. Cell Biol.* 16, 533-544. 10.1038/nrm4032 26285679 PMC4744649

[JCS182188C25] Jungmichel S., Clapperton J. A., Lloyd J., Hari F. J., Spycher C., Pavic L., Li J., Haire L. F., Bonalli M., Larsen D. H. et al. (2012). The molecular basis of ATM-dependent dimerization of the Mdc1 DNA damage checkpoint mediator. *Nucleic Acids Res.* 40, 3913-3928. 10.1093/nar/gkr1300 22234878 PMC3351161

[JCS182188C26] Kakarougkas A., Ismail A., Chambers A. L., Riballo E., Herbert A. D., Künzel J., Löbrich M., Jeggo P. A. and Downs J. A. (2014). Requirement for PBAF in transcriptional repression and repair at DNA breaks in actively transcribed regions of chromatin. *Mol. Cell* 55, 723-732. 10.1016/j.molcel.2014.06.028 25066234 PMC4157577

[JCS182188C27] Kumar M. S., Pester R. E., Chen C. Y., Lane K., Chin C., Lu J., Kirsch D. G., Golub T. R. and Jacks T. (2009). Dicer1 functions as a haploinsufficient tumor suppressor. *Genes Dev.* 23, 2700-2704. 10.1101/gad.1848209 19903759 PMC2788328

[JCS182188C28] Lan L., Ui A., Nakajima S., Hatakeyama K., Hoshi M., Watanabe R., Janicki S. M., Ogiwara H., Kohno T., Kanno S.-i. et al. (2010). The ACF1 complex is required for DNA double-strand break repair in human cells. *Mol. Cell* 40, 976-987. 10.1016/j.molcel.2010.12.003 21172662

[JCS182188C29] Lukas C., Melander F., Stucki M., Falck J., Bekker-Jensen S., Goldberg M., Lerenthal Y., Jackson S. P., Bartek J. and Lukas J. (2004). Mdc1 couples DNA double-strand break recognition by Nbs1 with its H2AX-dependent chromatin retention. *EMBO J.* 23, 2674-2683. 10.1038/sj.emboj.7600269 15201865 PMC449779

[JCS182188C30] Lukas C., Bartek J. and Lukas J. (2005). Imaging of protein movement induced by chromosomal breakage: tiny ‘local’ lesions pose great ‘global’ challenges. *Chromosoma* 114, 146-154. 10.1007/s00412-005-0011-y 15988581

[JCS182188C31] Lukas J., Lukas C. and Bartek J. (2011). More than just a focus: the chromatin response to DNA damage and its role in genome integrity maintenance. *Nat. Cell Biol.* 13, 1161-1169. 10.1038/ncb2344 21968989

[JCS182188C32] Melander F., Bekker-Jensen S., Falck J., Bartek J., Mailand N. and Lukas J. (2008). Phosphorylation of SDT repeats in the MDC1 N terminus triggers retention of NBS1 at the DNA damage-modified chromatin. *J. Cell Biol.* 181, 213-226. 10.1083/jcb.200708210 18411307 PMC2315670

[JCS182188C33] Michalik K. M., Bottcher R. and Forstemann K. (2012). A small RNA response at DNA ends in Drosophila. *Nucleic Acids Res.* 40, 9596-9603. 10.1093/nar/gks711 22848104 PMC3479179

[JCS182188C34] Nittner D., Lambertz I., Clermont F., Mestdagh P., Köhler C., Nielsen S. J., Jochemsen A., Speleman F., Vandesompele J., Dyer M. A. et al. (2012). Synthetic lethality between Rb, p53 and Dicer or miR-17–92 in retinal progenitors suppresses retinoblastoma formation. *Nat. Cell Biol.* 14, 958-965. 10.1038/ncb2556 22864477

[JCS182188C35] Pryde F., Khalili S., Robertson K., Selfridge J., Ritchie A.-M., Melton D. W., Jullien D. and Adachi Y. (2005). 53BP1 exchanges slowly at the sites of DNA damage and appears to require RNA for its association with chromatin. *J. Cell Sci.* 118, 2043-2055. 10.1242/jcs.02336 15840649

[JCS182188C36] Ravi A., Gurtan A. M., Kumar M. S., Bhutkar A., Chin C., Lu V., Lees J. A., Jacks T. and Sharp P. A. (2012). Proliferation and tumorigenesis of a murine sarcoma cell line in the absence of DICER1. *Cancer Cell* 21, 848-855. 10.1016/j.ccr.2012.04.037 22698408 PMC3385871

[JCS182188C37] Shiloh Y. (2006). The ATM-mediated DNA-damage response: taking shape. *Trends Biochem. Sci.* 31, 402-410. 10.1016/j.tibs.2006.05.004 16774833

[JCS182188C38] Stracker T. H. and Petrini J. H. j. (2011). The MRE11 complex: starting from the ends. *Nat. Rev. Mol. Cell Biol.* 12, 90-103. 10.1038/nrm3047 21252998 PMC3905242

[JCS182188C39] Stucki M., Clapperton J. A., Mohammad D., Yaffe M. B., Smerdon S. J. and Jackson S. P. (2005). MDC1 directly binds phosphorylated histone H2AX to regulate cellular responses to DNA Double-strand breaks. *Cell* 123, 1213-1226. 10.1016/j.cell.2005.09.038 16377563

[JCS182188C39a] Wang Q. and Goldstein M. (2016). Small RNAs recruit chromatin modifying enzymes MMSET and Tip60 to reconfigure damaged DNA upon double-strain break and facilitate repair. *Cancer Res.* pii: canres.2334.2015 [Epub] doi:10.1158/0008-5472.CAN-15-2334 10.1158/0008-5472.CAN-15-2334 26822153

[JCS182188C40] Wei W., Ba Z., Gao M., Wu Y., Ma Y., Amiard S., White C. I., Danielsen J. M., Yang Y.-G. and Qi Y. (2012). A role for small RNAs in DNA double-strand break repair. *Cell* 149, 101-112. 10.1016/j.cell.2012.03.002 22445173

[JCS182188C41] White E., Schlackow M., Kamieniarz-Gdula K., Proudfoot N. J. and Gullerova M. (2014). Human nuclear Dicer restricts the deleterious accumulation of endogenous double-stranded RNA. *Nat. Struct. Mol. Biol.* 21, 552-559. 10.1038/nsmb.2827 24814348 PMC4129937

